# Characterizing newborn and older infant entries into care in England between 2006 and 2014

**DOI:** 10.1016/j.chiabu.2020.104760

**Published:** 2020-11

**Authors:** Rachel J. Pearson, Matthew A. Jay, Melissa O’Donnell, Linda Wijlaars, Ruth Gilbert

**Affiliations:** a Population, Policy and Practice Research and Teaching Department, University College London Great Ormond Street Institute of Child Health, London, United Kingdom; b Telethon Kids Institute, University of Western Australia, Perth, Western Australia, Australia; c Australian Centre for Child Protection, University of South Australia, Adelaide, South Australia, Australia

**Keywords:** Entry into care, Infancy, Latent class analysis, Longitudinal data, Out-of-court arrangements

## Abstract

**Background:**

The risk of entry to state care during infancy is increasing, both here in England and abroad, with most entering within a week of birth (‘newborns’). However, little is known about these infants or of their pathways through care over early childhood.

**Objective:**

To characterize infant entries to care in England.

**Participants and setting:**

All children in England who first entered care during infancy, between April 2006 and March 2014 (n = 42,000).

**Methods:**

We compared sociodemographic and care characteristics for infants entering care over the study period by age at first entry (newborn: <1wks, older infant 1-51wks). Among those who entered before April 2010, we further characterized care over follow-up (i.e. 4 years from first entry) and employed latent class analysis to uncover any common pathways through care.

**Results:**

Almost 40 % of infants first entered care as a newborn. Most infants first entered care under s 20 arrangements (i.e. out-of-court, 60 % of newborns vs 47 % of older infants). Among infants entering before April 2010, most were adopted over follow-up (60 % vs 37 %), though many were restored to parental care (20 % vs 32 %) or exited care to live with extended family (13 % vs 19 %). One in six infants (17.7 %) had particularly unstable care trajectories over early childhood, typified by three or more placements or failed reunification.

**Conclusions:**

Evidence-based strengthening of pre-birth social work support is needed to improve preventive interventions before birth, to more effectively target infant placement into care. Linkages between child protection records and information on parents are needed to inform preventive strategies.

## 1 Introduction

### 1.1 Infancy, child maltreatment, and entry into care

Child maltreatment (i.e. abuse and neglect) during infancy (<12 months old), a crucial time for child development, is associated with poorer physical, intellectual, and behavioral growth (Ward, Brown, & Westlake, 2012). Good attachment behaviors between an infant and their caregiver are critical for developing relationships, coping with stress, and parenting in later life. In contrast, poor attachment behaviors, such as inconsistent or abusive caregiving or neglect are associated with severe and long-lasting emotional and behavioral problems (Naughton et al., 2013). Infants are particularly vulnerable to maltreatment, with their physical and developmental immaturity placing them at greater risk of impacts on their development, serious injury, and mortality (Child Welfare Information Gateway, 2018; Sidebotham et al., 2016). Maltreatment can also occur in utero, for example, harm to fetal development from maternal substance misuse or domestic violence (O’Connor et al., 2020; O’Leary, Lawrence, Hafekost, Zubrick, & Bower, 2020).

### 1.2 Infant entry into care

Infants constitute a large proportion of children who enter care and the annual rate of infant entry into care is rising in several high-income settings (Children’s Bureau, n.d.; Department for Education, 2019b; Marsh, Browne, Taylor, & Davis, 2017; O’Donnell, Taplin, Marriott, Lima, & Stanley, 2019; Oranga Tamariki, 2019; Woods & Henderson, 2018). There is emerging evidence that increases among infants are largely driven by a rise in newborn entries into care (i.e. < 7 days old) (Bilson & Bywaters, 2020; Manitoba Liberal Caucas, 2019). These emerging trends have received intense media focus, particularly in Australia and Aotearoa New Zealand where babies born to Indigenous mothers are disproportionately affected (Keddell, 2019; New Zealand Family Violence Clearing House, 2019; O’Donnell et al., 2019; Oranga Tamariki, 2019; Wahlquist, 2019).

Many infant entries into care follow child protection concerns identified during pregnancy. For example, parents in such cases in England are typically assessed, before birth, and may be offered services such as parenting courses or targeted support (e.g. for mental health problems, substance misuse, and domestic violence) to improve parenting capacity and to reduce further need for child protection. Nevertheless, in several countries, including England, parents cannot legally be compelled to take part in assessments or to engage with services (Hestbæk, Höjer, Pösö, & Skivenes, 2020; Masson & Dickens, 2015). Furthermore, pregnancy affords little time to make and evidence the often-significant improvements to parenting capacity necessary to protect a child from harm and to promote healthy development. Rehabilitation of parenting capacity should also be able to occur in time to promote healthy attachment and child development over infancy.

### 1.3 The context in England

#### 1.3.1 Routes of entry into care in England

In England, the responsibility for placing children into care is devolved to the 152 local authorities under the Children Act 1989 (UK Government, 1989). When a child is at risk of significant harm, local authorities must apply to the family court to initiate public family law proceedings (‘care proceedings’ under section 31 of the Act) to place a child into care. Children can also enter care via out-of-court arrangements (‘s 20 arrangements’ under section 20 of the Act), which are applicable only where the child appears to have no caregivers (e.g. abandoned, lost or orphaned), or the birth parents are prevented from providing suitable accommodation or care and do not object to the child being accommodated by the local authority. Most children are placed with stranger (i.e. unrelated) foster carers; others are placed with extended family (i.e. kinship foster) or in family or mother-baby placements (Department for Education, 2019b). Older children may be placed in residential care homes, secure units, or in (semi-)independent accommodation (Department for Education, 2019b, 2020). A small number of children ‘in care’ in England remain at home under local authority supervision (Department for Education, 2019b). Most children are in care only for a short period, with a median length of stay of 4 months and a median of 2 placements between birth to 18 years old (Mc Grath-Lone, Harron, Dearden, & Gilbert, 2020).

#### 1.3.2 Responding to child protection concerns about unborn children

**Fig. 1:**
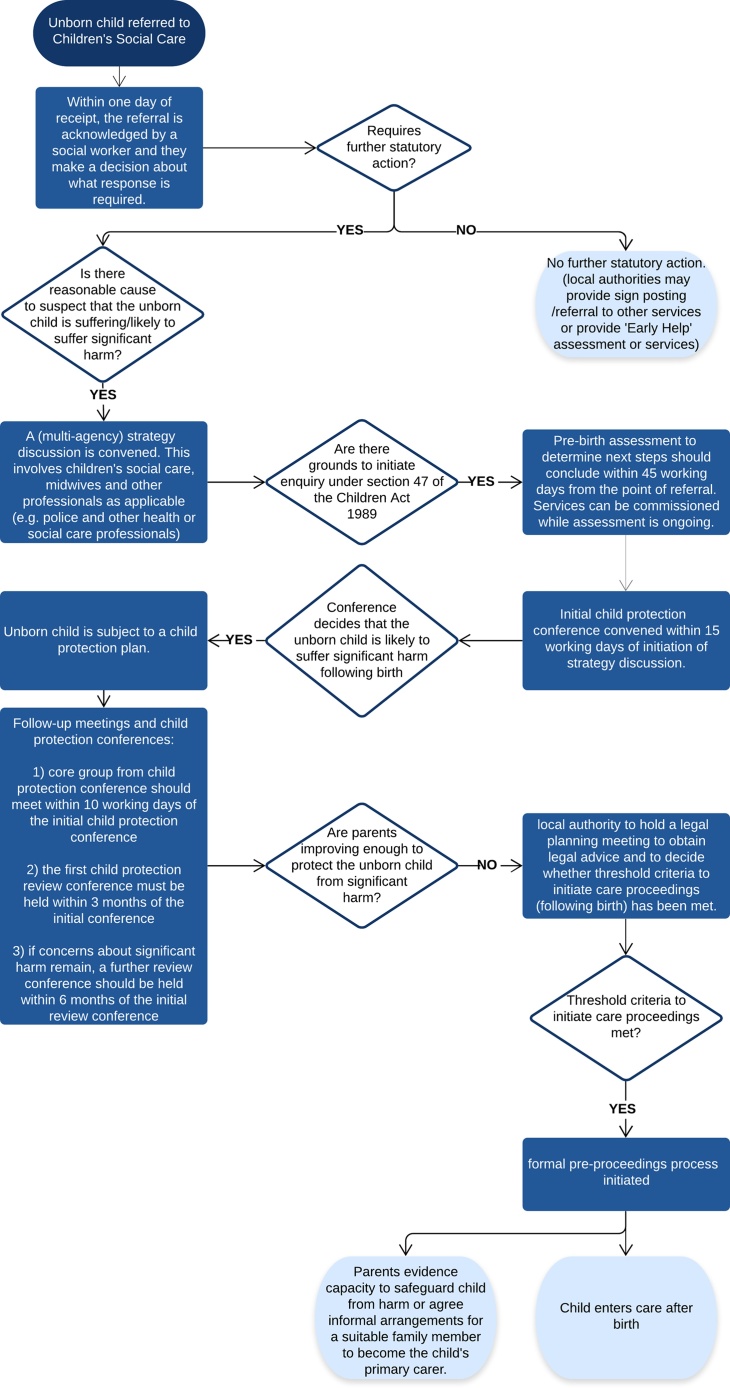
Outlining statutory pre-birth child protection procedures in England.

Identifying child protection concerns during pregnancy provides a unique opportunity for health and social care professionals to engage parents and reduce risks before a child is born. Fig. 1 outlines the statutory child protection procedure in England that all local authorities must follow when responding to child safeguarding concerns, whether the child is born or not (HM Government, 2018). Pre-birth assessments are a crucial part of these procedures and are used to identify safeguarding risks, and to connect parents to support services (Department for Education, 2014; HM Government, 2018). If concerns are substantiated, a child protection plan may be drawn up before birth, with 2.3 % of children subject to a child protection plan on 31st March 2019 not yet born (Department for Education, 2019a). If it becomes clear that the parents cannot make improvements to parenting capacity in time to support key development milestones over infancy (Brown & Ward, 2013), the local authority may instigate care proceedings (HM Government, 2018).

As legal personhood is realized only once a child is born alive under English law (*Re F (in Utero)*, 1988), care proceedings cannot commence until after birth. In the intervening period, a formal pre-proceedings process will be initiated and parents become eligible, in principle, for free legal advice (Department for Education, 2014). However recent evidence suggests that outside of London in particular, finding legal representation may be difficult (Mason & Broadhurst, 2020).

Most local authorities have built upon national statutory guidance by developing local protocols tailored to referrals involving unborn children, which contributes to variation in the quality of guidance and pre-birth child protection practice across England (Lushey, Barlow, Rayns, & Ward, 2018). There is also an abundance of qualitative evidence suggesting many practitioners have insufficient guidance on these pre-birth procedures, resulting in delayed pre-birth assessments and missed opportunities for early intervention (Honey, Miceli, & Mayes, 2019; Lushey et al., 2018; Mason, Robertson, & Broadhurst, 2019).

#### 1.3.3 Recent evidence on infant entries into care in England

The Department for Education produce annual statistics on all children entering local authority care in England, stratified by age group (including <1 year old); infants account for around 20 % of entries into care each year (Department for Education, 2019b). More and more of these infants are entering care soon after birth, with the number of newborns entering care (per 10,000 live-born) rising from 26 per 10,000–48 per 10,000 between 2007/08 and 2017/18 (Bilson & Bywaters, 2020); around half of newborn entrants initially entered care under s 20 arrangements. Half of newborn entrants were later placed for adoption, while 35 % exited care to live with extended family. Previous research using administrative family court data reported that newborns involved in care proceedings, most of whom enter care, were less likely than older infants to be placed with parents or extended family (Broadhurst et al., 2018). Furthermore, around 50 % of newborns involved in care proceedings had an older sibling previously subject to proceedings, highlighting that many newborn entries into care are driven by ‘recurrent’ parents (Broadhurst et al., 2018, 2017). Both newborn rates of entry into care and involvement in care proceedings varied considerably by region and were highest in the North East and lowest in London (Bilson & Bywaters, 2020; Broadhurst et al., 2018).

Evidence remains scarce on infants’ pathways through care, despite the importance of early childhood for developmental milestones. Evidence from one large local authority found that children aged 0–2 years old had very different pathways through care and may be less likely to be restored to parental care, than older children (Neil, Gitsels, & Thoburn, 2019; Neil, Gitsels, & Thoburn, 2019). It is also unclear whether increased newborn entry into care is partly driven by earlier placement of infants into care who would have otherwise entered care later in infancy. Finally, more research is needed to compare sociodemographic and care characteristics between infants entering care as newborns and those entering later in infancy, for example, to better understand whether newborns are as likely to be placed with kin or to be restored to parental care as older infants. Addressing these gaps will inform future studies seeking to understand whether enough is being done to support parenting and avoid unnecessary infant entry into care in England, to guide evidence-based policy and practice.

### 1.4 Objectives of this study

We used longitudinal, episode-level data from the Department of Education containing information on all child episodes in care in England between April 2006 and March 2014 (Mc Grath-Lone, Harron, Dearden, Nasim, & Gilbert, 2016). Specifically, we had three objectives:

1) To understand whether increases to newborn (<1wk old) entry into care have led to decreased entry among some older infant age groups (1–3wks; 4-12wks; 13-25wks; 26–38wks; 39–51wks).
2) To explore whether sociodemographic and care characteristics differ between children who enter care as newborns (<1wk old) and those who enter later in infancy (1–51wks);
3) To identify and examine clusters of infants with have similar pathways through care over early childhood.

## 2 Materials and methods

### 2.1 Study cohort

We included all children who first entered local authority care in England between 1st April 2006 and 31st March 2014 under non-respite arrangements and were under one year old at entry (n = 42,000). We restricted the cohort to include only children first entering care before 1st April 2010 when examining differences in early childhood care experiences, to allow for four years of follow-up from age of first entry into care (n = 18,440). This sub-cohort excluded children who died or transferred to the care of another local authority over follow-up, as they had incomplete data (n = 230). We also lack information on children placed for adoption who re-enter care as these children receive a new unique identifier in the data following adoption.

### 2.2 Data sources

#### 2.2.1 Children Looked After (CLA) return

We used a de-identified extract of the Children Looked After return (CLA) to create a longitudinal cohort of children born after 31^st^ March 2005 who first entered care during infancy (<52wks old) between 1st April 2006 and 31st March 2014; (Mc Grath-Lone et al., 2016) we used CLA data between 1st April 2005 and 31st March 2006 to identify and excluded children who entered care over this period. We additionally excluded children who first entered care under short-term breaks (i.e. a series of short-term placements often for children with complex health needs, also known as respite arrangements) as they are likely to have very different care needs than the remainder of children in care. Further information on cohort selection is available in the supplementary appendix (Fig. S1).

### 2.3 Measures

#### 2.3.1 Sociodemographic characteristics at first entry to care

These included sex, ethnic group, age at first entry, and local authority-level deprivation status (using the 2010 Index of Multiple Deprivation quintiles) (Ministry of Housing Communities & Local Government, 2011).

#### 2.3.2 Care characteristics at first entry to care

These included legal route of entry into care (e.g. Care Order, s 20 arrangement, emergency protection, etc.), placement type (e.g. stranger foster care, kinship foster care, placement in a healthcare setting, etc.) and category of need (e.g. abuse and neglect, parental disability, absent parenting, etc.).

#### 2.3.3 Care characteristics over early childhood

Among our sub-cohort, we looked at the following outcomes captured over the four-year follow-up period: 1) placement type, 2) type of exit from care (e.g. adoption, restoration to parental care, etc.), 3) number of care placements, and 4) duration in care.

Full definitions of measures used are available in the supplementary appendix.

### 2.4 Descriptive and statistical analyses

We calculated the annual rates (April-March) of entry into care by age at first entry (newborn: <1wk old, 1–3 wks, 4–11 wks, 12–25 wks, 26–38 wks, 39–51 wks) per 10,000 live births (Office for National Statistics, 2017).

We compared our derived annual rates for newborn (per 10,000 live births) and infant (per 10,000 infants) entry into care between 2006/07 and 2013/14 with rates derived from figures in research articles or government statistics from Australia (Overall and in New South Wales (NSW)) (Australian Institute of Health & Welfare, 2019a; O’Donnell et al., 2019), and Aotearoa New Zealand (Oranga Tamariki, 2019), up to 2017/18. To compare rates in more recent years, we additionally included the 2017/18 newborn rate of entry into care from Bilson & Bywaters, 2020, and derived infant rate of entry into care between 2014/15 and 2017/18 using Department for Education reported statistics on the number of infants entering care each year (Department for Education, 2019a). These countries and regions for comparison were chosen based on the availability of published data (for more details on data used for these country comparisons, see Table S1 of the supplementary appendix). Although there are no whole-population published figures on newborn entries into care in the USA, we compared rates of infant entry into care from the USA with England and the other selected countries (Children’s Bureau, 2007-2018). With high rates of risk factors for child maltreatment and placement into state care, the USA serves as an important comparator (Gilbert et al., 2012).

We produced descriptive statistics for sociodemographic and case characteristics at first entry among our full cohort and for early childhood outcomes over follow-up among our sub-cohort. These were presented by year (April-March) and age group (newborn: <1wk and older infant: 1-51wk) at first entry.

To further explore all infants’ pathways through care among our sub-cohort, we used latent class analysis (LCA) to uncover any groupings of children with similar care characteristics over the four-year follow-up period. LCA can be a useful tool to categorize people with similar attributes and has been used previously with administrative child protection records (Eastman, Mitchell, & Putnam-Hornstein, 2016; Mc Grath-Lone, 2017). Informed by prior research using LCA with CLA data to describe pathways through care (Mc Grath-Lone, 2017), we created eight indicators for inclusion in our LCA models: (1) first placed under a s 20 arrangement; (2) ever in care under a Care or Placement Order over follow-up; (3) ever assigned category of need ‘Abuse or Neglect’ over follow-up; (4) in care for less than one year over follow-up; (5) more than two placements in care over follow-up; (6) adopted over follow-up; (7) at least one exit and re-entry into care over follow-up; and (8) ever placed with family (incl. mother-baby placement) over follow-up. Our LCA was carried out using the poLCA package in R v3.6.2 (Linzer & Lewis, 2011).

To improve the likelihood of our models converging to the global maximum likelihood solution, rather than to a local maxima, we increased the number of randomly-selected starting values with the number of classes (Collins & Lanza, 2010). We tried models with 1–7 latent class, though our seven-class LCA model did not converge. To assess model fit for our 1–6 class models, we used the likelihood-ratio G^2^ statistic. We compared relative model fit using the Akaike information criterion (AIC) and the Bayesian information criterion (BIC), as well as by the interpretability of each model’s latent groupings. Based on these checks, we selected the six-class LCA model as our final model. We then assigned children to latent groups using maximum probability assignment (i.e. children were assigned to the latent group for which their probability of group membership was largest) and performed several checks on the model posterior probabilities which indicated that the six latent groupings were well defined from one another. Further details on these checks are available in the supplementary appendix.

### 2.5 Ethical approval and statistical disclosure rules

Data were de-identified and approved for research before we received them, therefore we did not require ethical approval. Following the Department for Education’s statistical disclosure rules, all counts displayed and used to calculate rates and percentages were rounded to the nearest 10 and counts between 1–9 were censored.

## 3 Results

### 3.1 Rates of infant entry into care in England between April 2006 and March 2014, by age of first entry

**Fig. 2:**
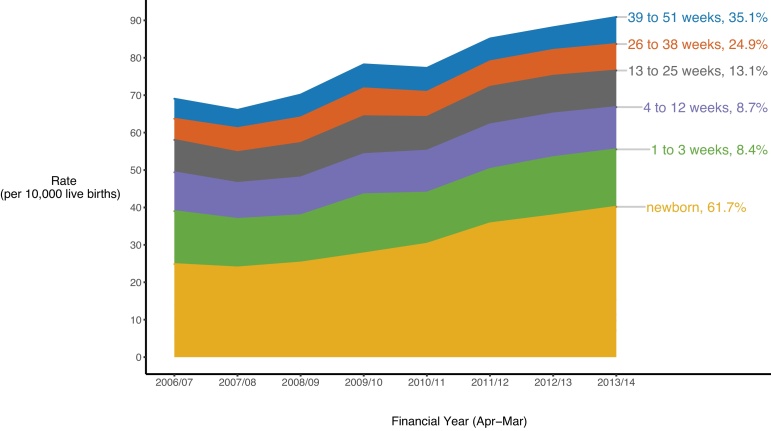
Cumulative annual (Apr-Mar) rates of infant entry into care per 10,000 live births in England, by age (weeks), from 2006/07–2013/14, and the percentage increase in rates over this period.

Among the 42,000 children who first entered care during infancy between April 2006 and March 2014 for non-respite care; 16,550 (39.4 %) first entered as a newborn. The annual rates of entry into care rose in all six infant age groups over the study period, though increases were steepest among newborns (Fig. 2). Increases to the rate of newborn entry into care accounted for 70.2 % of the 31.6 % increase to the rate of all infant entries into care between 2006/07 and 2013/14.

### 3.2 Comparing rates of infant and newborn entry into care between countries

**Fig. 3:**
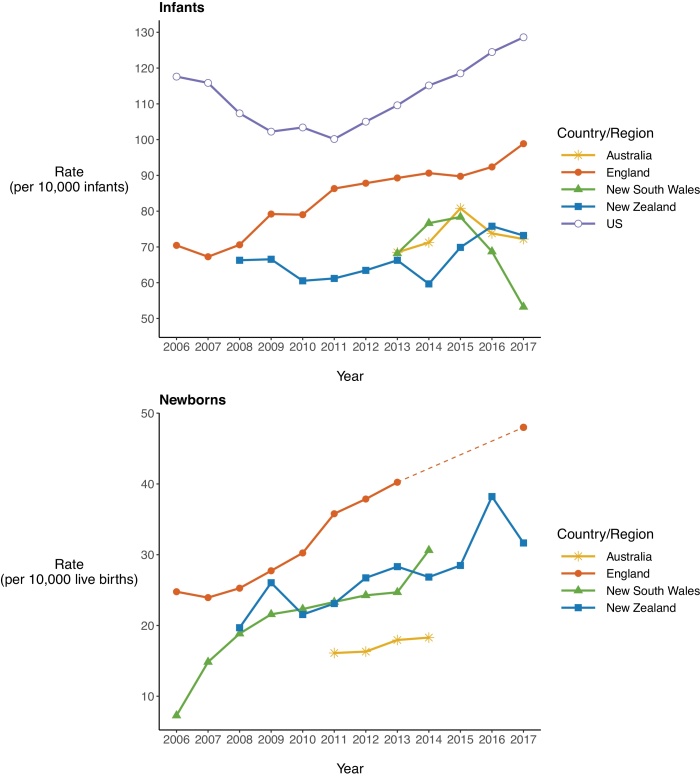
Comparing rates of infant (top) and newborn (bottom) entry into care between England and other settings (2006–2017). Note: the dotted line demonstrates the linear trajectory between our study 2013/14 rate of newborn entry into care (per 10,000 livebirths) and Bilson & Bywater’s 2017/18 rate.

Among countries with available data, the US had the largest rates of infant entry into care (Fig. 3, top). However, England has far higher rates of infant entry into care than Australia and Aotearoa New Zealand. While data on newborn entries into care were unavailable for the US, England again had much higher rates than Australia (overall), NSW, and Aotearoa New Zealand. Newborn entry into care rose over time in each of these settings (Fig. 3, bottom). Data from Australia and the US include only children in out-of-home care. However, few infants (3.4 %) placed into care in England over the study period were placed at home with parents initially (Table 1).

**Table 1:** Sociodemographic and care characteristics among children who first entered care in England during infancy between 1st April 2006 and 31st March 2014, stratified by age at entry (newborn: <1wk, older infant: 1-51wks) and year of entry (n = 42,000).

Characteristics at first entry into care	Time and age at first entry into care							
	2006/07–2007/8		2008/09–2009/10		2010/11–2011/12		2012/13–2013/14	
	Newborn	older infant	newborn	older infant	newborn	older infant	newborn	older infant
**Number of children**	3140	5560	3560	6400	4540	6630	5300	6850

**Female (%)**	1530 (48.7)	2620 (47.1)	1750 (49.2)	3010 (47.0)	2250 (49.6)	3120 (47.1)	2580 (48.7)	3250 (47.4)

**Ethnic Group (%)**								
White	2430 (77.4)	4180 (75.2)	2760 (77.5)	4910 (76.7)	3550 (78.2)	5070 (76.5)	4030 (76)	5060 (73.9)
Mixed	400 (12.7)	720 (12.9)	410 (11.5)	760 (11.9)	540 (11.9)	810 (12.2)	610 (11.5)	830 (12.1)
Black	150 (4.8)	380 (6.8)	180 (5.1)	380 (5.9)	170 (3.7)	350 (5.3)	160 (3.0)	330 (4.8)
Asian	130 (4.1)	180 (3.2)	120 (3.4)	190 (3.0)	110 (2.4)	210 (3.2)	140 (2.6)	240 (3.5)
Other	30 (1.0)	100 (1.8)	50 (1.4)	110 (1.7)	60 (1.3)	100 (1.5)	90 (1.7)	160 (2.3)
Missing	0	0	50 (1.4)	70 (1.1)	110 (2.4)	100 (1.5)	260 (4.9)	240 (3.5)

**Average age at first entry, days (median [25^th^, 75^th^ percentile])**	2.5 [1.0, 4.0]	71.0 [19.0, 186.0]	3.0 [1.0, 4.0]	84.0 [20.0, 197.0]	3.0 [1.0, 4.0]	80.0 [21.0, 194.0]	3.0 [1.0, 4.0]	79.0 [19.0, 195.0]

**Local Authority IMD 2010 quintile**								
1 – most deprived	320 (10.2)	670 (12.1)	420 (11.8)	820 (12.8)	470 (10.4)	800 (12.1)	550 (10.4)	830 (12.1)
2	660 (21)	1340 (24.1)	800 (22.5)	1550 (24.2)	1080 (23.8)	1640 (24.7)	1220 (23)	1600 (23.4)
3	510 (16.2)	940 (16.9)	590 (16.6)	1100 (17.2)	810 (17.8)	1140 (17.2)	920 (17.4)	1180 (17.2)
4	770 (24.5)	1160 (20.9)	850 (23.9)	1320 (20.6)	1100 (24.2)	1410 (21.3)	1290 (24.3)	1490 (21.8)
5 - least deprived	870 (27.7)	1430 (25.7)	890 (25)	1580 (24.7)	1070 (23.6)	1620 (24.4)	1280 (24.2)	1700 (24.8)

**Most common primary category of need (%)**^a^								
Abuse and Neglect	2050 (65.3)	3840 (69.1)	2340 (65.7)	4460 (69.7)	3170 (69.8)	4650 (70.1)	3630 (68.5)	4790 (69.9)
Parental illness or disability	150 (4.8)	370 (6.7)	170 (4.8)	410 (6.4)	210 (4.6)	430 (6.5)	240 (4.5)	340 (5.0)
Family in acute stress	280 (8.9)	480 (8.6)	270 (7.6)	490 (7.7)	270 (5.9)	400 (6.0)	310 (5.8)	450 (6.6)
Family dysfunction	350 (11.1)	650 (11.7)	530 (14.9)	830 (13)	700 (15.4)	950 (14.3)	930 (17.5)	1090 (15.9)
Absent parenting	300 (9.6)	140 (2.5)	220 (6.2)	120 (1.9)	160 (3.5)	130 (2.0)	140 (2.6)	90 (1.3)

**Most common legal status (%)**								
Care Order	830 (26.4)	2180 (39.2)	1050 (29.5)	2350 (36.7)	1510 (33.3)	2590 (39.1)	1950 (36.8)	2770 (40.4)
Emergency child protection^b^	360 (11.5)	780 (14.0)	390 (11.0)	940 (14.7)	430 (9.5)	850 (12.8)	490 (9.2)	890 (13.0)
Section 20 arrangements	1950 (62.1)	2600 (46.8)	2120 (59.6)	3110 (48.6)	2590 (57.0)	3170 (47.8)	2860 (54.0)	3190 (46.6)

**Most common placement type (%)**								
Stranger foster carer	2510 (79.9)	4050 (72.8)	2810 (78.9)	4680 (73.1)	3490 (76.9)	4650 (70.1)	4060 (76.6)	4780 (69.8)
Kinship foster carer	100 (3.2)	510 (9.2)	150 (4.2)	690 (10.8)	230 (5.1)	870 (13.1)	310 (5.8)	950 (13.9)
Healthcare setting	360 (11.5)	400 (7.2)	350 (9.8)	380 (5.9)	480 (10.6)	460 (6.9)	590 (11.1)	480 (7.0)
With parents at home	90 (2.9)	420 (7.6)	100 (2.8)	410 (6.4)	110 (2.4)	400 (6.0)	90 (1.7)	350 (5.1)
Family/mother-baby placement	60 (1.9)	140 (2.5)	120 (3.4)	190 (3.0)	220 (4.8)	200 (3.0)	230 (4.3)	250 (3.6)

a The primary category of need is hierarchical, with ‘Abuse or Neglect’ the highest category in the hierarchy (i.e. if multiple categories of need are identified then only the highest in the hierarchy will be recorded). These categories are presented in order, with the highest at the top.

b Entry under emergency protection orders (child can enter care for up to 8 days) and police protection powers (child can enter care for up to 72 h).

### 3.3 Trends in characteristics at first entry to care among infants entering care in England between April 2006 and March 2014

Table 1 describes the characteristics at first entry to care among the 42,000 children who first entered care during infancy between April 2006 and March 2014 There were roughly equal numbers of female and male infants entering care and over three quarters were White. On average, newborns entered care 1–4 days following their birth, whereas most first entering care later in infancy did so between 1wk-6mo following their birth. Most infants first entered care due to experience or risk of abuse or neglect.

Newborn infants were more likely to enter care under s 20 arrangements than older infants, though the use of s 20 for newborn entries to care appeared to decline over time (62.0 % between April 2006 and March 2008 vs 54.0 % between April 2012 and March 2014). A smaller proportion of newborns first entered care under emergency child protection such as police protection powers or emergency protection orders compared to older infant entrants.

Most infants were initially placed into stranger foster care, though initial placement with kinship foster carers (typically extended family) or in family/mother-baby units increased slightly over the study period among all infant entrants. Fewer newborns were placed with kinship foster carers or with their parents compared to older infants and one in ten newborn entrants spent their first placement in a healthcare setting.

Further investigation into ethnic inequalities in the rate of newborn entry into care (per 10,000 livebirths) revealed that rates were much lower among babies with Asian ethnicity recorded than babies with White ethnicity recorded (Table S4, supplementary appendix). While rates in the observed data were highest for Other (incl. Mixed) ethnicity, followed by White then Black ethnicity, there was a large amount of missing data for the denominator (i.e. livebirths) which could result in higher rates among Black and Other ethnicity compared to White ethnicity. Further research is needed to better understand the systemic causes of these differences.

### 3.4 Trends in characteristics within four years from first entry to care among infants entering care in England between April 2006 and March 2010

Among infants in the cohort who entered care before April 2010 (n = 18,440), 92.4 % exited care at least once within four years from their first entry (Table 2). Three-fifths (58.2 %) of newborn entrants were adopted over follow-up, compared to 37.2 % of older infant entrants. Conversely, newborns were less likely to be restored to parental care over follow-up than older infants (19.7 % vs 31.6 %). Newborns were also less likely to be placed with their parents at home (13.9 % vs 19.6 %) or to exit care to live with extended family (12.8 % vs 18.4 %). Few infants of any age (7.3 %, 7.8 % of newborns, 6.9 % of older infants) were ever placed in a mother-baby or family placement over follow-up. Most infants (70.8 %) spent more than 11 months in care over the four-year follow-up and 37.6 % had three or more placements in care.

**Table 2:** Care characteristics over four years from first entry among children who first entered care in England during infancy between 1st April 2006 and 31st March 2010, stratified by age at entry (newborn: <1wk, older infant: 1-51wks) and year of entry (n = 18,440).

	Time and age at first entry into care			
	2006/07–2007/8		2008/09–2009/10	
	newborn	older infant	newborn	older infant
**Number of children**	3140	5560	3560	6400

**Number of children after applying exclusions (%)**^a^	3110 (99.0)	5480 (98.6)	3530 (99.2)	6320 (98.8)

**Types of placement over follow-up (%)**				
At home (e.g. under a supervision order)	440 (14.1)	1130 (20.6)	490 (13.9)	1210 (19.1)
Family/mother-baby placement	220 (7.1)	350 (6.4)	300 (8.5)	480 (7.6)
Kinship foster carers	360 (11.6)	1080 (19.7)	420 (11.9)	1380 (21.8)
Placed for adoption	1920 (61.7)	2270 (41.4)	2090 (59.2)	2500 (39.6)

**Exits from care over follow-up (%)**				
Any exit	2950 (94.9)	4990 (91.1)	3310 (93.8)	5790 (91.6)
Exited but later re-entered care	140 (4.5)	530 (9.7)	200 (5.7)	680 (10.8)
Restored to parental care	600 (19.3)	1780 (32.5)	720 (20.4)	2000 (31.6)
Exit to live with extended family	380 (12.2)	920 (16.8)	480 (13.6)	1280 (20.3)
Exit to adoption	1870 (60.1)	2120 (38.7)	2030 (57.5)	2330 (36.9)

**Duration in care over follow-up (%)**				
Median duration (years) [25 %, 75 % quantile]	1.51 [0.96, 2.16]	1.55 [0.71, 2.41]	1.63 [1.06, 2.31]	1.64 [0.83, 2.55]
0 to 5 months	340 (10.9)	1030 (18.8)	340 (9.6)	1070 (16.9)
6 to 11 months	490 (15.8)	800 (14.6)	470 (13.3)	820 (13.0)
12 to 23 months	1360 (43.7)	1720 (31.4)	1470 (41.6)	1940 (30.7)
24 or more months	920 (29.6)	1920 (35.0)	1250 (35.4)	2480 (39.2)

**Number of placements over follow-up (%)**				
1-2	2010 (64.6)	3370 (61.5)	2260 (64.0)	3860 (61.1)
3-4	910 (29.3)	1720 (31.4)	1060 (30.0)	1960 (31.0)
5+	200 (6.4)	390 (7.1)	210 (5.9)	490 (7.8)

a All subsequent percentages are derived using the number of children after applying exclusions.

### 3.5 Patterns in type, length and stability of care over early childhood among children first entering care during infancy between April 2006 and March 2010

Table 3 shows the six classes of infant pathways through care over early childhood, identified in the latent class analysis.

**Table 3:** Comparing age at first entry, latent class model indicators, and reasons for last exit from care over follow-up among our six latent groups (n = 18,440).

	Group one (N = 2230, 12.1 %)	Group two (N = 710, 3.9 %)	Group three (N = 3160, 17.1 %)	Group four (N = 3560, 19.3 %)	Group five (N = 1030, 5.6 %)	Group six (N = 7740, 42.0 %)
**Newborn (<7 days old) at first entry into care (%)**	710 (31.8)	560 (78.9)	860 (27.2)	960 (27.0)	220 (21.4)	3330 (43.0)
**Average age at first entry, days (median [25^th^, 75^th^ percentile])**	15 [5, 102]	2 [1, 5]	60 [6, 197]	28 [6, 137]	82 [10, 202]	9 [3, 76]
**Latent class model indicators (%)**						
Ever assigned category of need 'Abuse or Neglect' over follow-up	1700 (76.2)	120 (16.9)	1690 (53.5)	2690 (75.6)	700 (68.0)	5820 (75.2)
First placed under Section 20	600 (26.9)	710 (100)	2710 (85.8)	710 (19.9)	830 (80.6)	4110 (53.1)
Ever in care under a Care or Placement Order over follow-up	2230 (100)	30 (4.2)	0	3560 (100)	820 (79.6)	7740 (100)
Ever placed with family (incl. mother-baby placement) over follow-up	2230 (100)	0	80 (2.5)	1570 (44.1)	200 (19.4)	0
In care for less than one year over follow-up	60 (2.7)	240 (33.8)	2660 (84.2)	2040 (57.3)	240 (23.3)	140 (1.8)
More than two placements in care over follow-up	2220 (99.6)	100 (14.1)	90 (2.8)	370 (10.4)	990 (96.1)	3180 (41.1)
Had at least one exit and re-entry into care over follow-up	260 (11.7)	0	170 (5.4)	140 (3.9)	980 (95.1)	0
Was adopted over follow-up	880 (39.5)	710 (100)	0	0	320 (31.1)	6430 (83.1)
**Reason for the last exit from care over follow-up (%)**						
Adoption	880 (39.5)	710 (100)	0	0	320 (31.1)	6430 (83.1)
Restored to parental care	340 (15.2)	0	2360 (74.7)	1100 (30.9)	170 (16.5)	90 (1.2)
To live with extended family	340 (15.2)	0	380 (12.0)	1460 (41.0)	190 (18.4)	580 (7.5)
Other exit^a^	150 (6.7)	0	300 (9.5)	490 (13.8)	30 (2.9)	40 (0.5)
Remained in care at end of follow-up	510 (22.9)	0	130 (4.1)	510 (14.3)	320 (31.1)	600 (7.8)

a Other includes: Child died, moved abroad, or the period of being looked after ceased for any other reason (i.e. unspecified).

#### 3.5.1 Latent group one: long stay with unstable placements, with at least one family placement (n = 2230, 12.1 %)

Most were in care due to abuse and neglect and spent longer than one year in care (97.3 %) within the four years following their first entry. Most had more than two placements over this time (99.6 %) and all were placed with their parents at least once, either at home or in a family placement (such as a mother-baby unit). The median age of first entry among this group was 15 days old.

#### 3.5.2 Latent group two: long stay with stable placements under s 20 arrangements, ending in adoption (n = 710, 3.9 %)

Few children were assigned to this group. Most were less than seven days old when they were first placed into care (78.9 %) and in care for reasons other than abuse and neglect (83.1 %). All children in this group first entered care under a s 20 arrangement. Further investigation revealed that 40.8 % first entered due to absent parenting (not reported). None were placed with their parents over follow-up. Most spent longer than a year in care (66.2 %) over follow-up and most had just one or two placements in care (85.9 %). All children were adopted within four years of entering care.

#### 3.5.3 Latent group three: short stay with stable placements under s 20 arrangements, restored to parental care (n = 3160, 17.1 %)

Most first entered care under a s 20 arrangement. Very few of these children were placed with their parents over follow-up (2.5 %). The majority spent less than one year in care over follow-up (84.2 %), over only one or two placements (97.2 %). Most children returned home within four years of entering care (74.7 %) and very few (5.4 %) subsequently re-entered care over follow-up. The median age of first entry among this group was 2 months old.

#### 3.5.4 Latent group four: stable placements under care or placement orders (n = 3560, 19.3 %)

This was the second largest group. Most were in care due to abuse and neglect (75.6 %) and all were subject to a care or placement order over follow up. Most had only one or two placements (89.6 %) and just over half spent less than one year in care (57.3 %). Very few children left and then subsequently re-entered care over follow-up (3.9 %). Most children exited care over follow-up, either returning home to parents (30.9 %) or exiting via an SGO or residence order (41.0 %). The median age of first entry among this group was 4 weeks old.

#### 3.5.5 Latent group five: Long stay, unstable placements, and re-entries, first under s 20 arrangements and later under care or placement orders (n = 1030, 5.6 %)

Most were in care due to abuse and neglect (68.0 %) and most first entered care under s 20 arrangements (80.6 %). However, approximately 80 % were later subject to a care or placement order. The majority spent more than one year in care over follow-up (76.7 %) and had more than two placements (96.1 %). Most exited and re-entered care at least once over follow-up (95.1 %). The median age of first entry among this group was approximately 12 weeks old.

#### 3.5.6 Latent group six: long stay under care or placement orders, ending in adoption (n = 7740, 42.0 %)

The was the largest group. Most were in care due to abuse and neglect (75.2 %) and all were subject to a care or placement order over follow-up. None were placed with their parents over follow-up and most spent longer than one year in care (98.2 %), with just over half having one or two placements (58.9 %). Most in this group were adopted within four years of first entering care (83.1 %). The median age of first entry among this group was 9 days old.

## 4 Discussion

### 4.1 Key results

The rate of entry into care increased among all infant age groups in England between 2006/07 and 2013/14. It is, therefore, unlikely that increased newborn entry to care over this period was driven by nationwide shifts in practice to place infants into care earlier. This is further supported by recent qualitative evidence that suggests increases were chiefly driven by greater demand and reduced availability of early family intervention services and other preventive support (Mason & Broadhurst, 2020).

Most infants first entered care via s 20 arrangements (newborn entrants: 60 % vs older infant entrants: 47 %), though this diminished over time as entry via care orders rose (Table 1). Most infants were subsequently adopted (newborn entrants: 60 % vs older infant entrants: 37 %), though large numbers exited care to live with extended family (13 % vs 19 %) or were restored to parental care (20 % vs 32 %) (Table 2). However, exits to kin, as well as placement with kin while under local authority care, were lower among newborns than among older infants, consistent with previous research using family court data (Broadhurst et al., 2018). This suggests that local authorities were more frequently unable to find (or did not pursue finding) suitable kinship carers for newborn entrants into care, compared to older infant entrants. Furthermore, fewer than 10 % infants were ever placed into mother-baby placements while in care, which include psychiatric mother-baby units, despite some evidence of a high prevalence of mental health problems among women whose children enter care (Griffiths et al., 2020).

We found considerable variation in the care pathways of infants entering care, particularly among those first entering care via s 20 arrangements, the most common route of entry into care among the cohort. For example, latent group three (s*hort stay with stable placements under s 20 arrangements, restored to parental care*) indicated that s 20 arrangements were often used to facilitate infants remaining with their birth parents long-term, the original intention for s 20 as outlined by the government’s 1987 white paper on child care law (Department of Health & Social Security, 1987). However, latent group five (*Long stay, unstable placements, and re-entries, first under s 20 arrangements and later under care or placement orders*) suggested that some children entering under s20 arrangements had at least one exit and re-entry to care, chiefly driven by abuse and neglect, indicating failed reunification. Finally, latent group two (*long stay with stable placements under s 20 arrangements, ending in adoption*) supported previous research that found s 20 arrangements were often used to facilitate ‘foster to adopt’ placements among newborns (Lynch, 2017).

Placement instability during early childhood, which is associated with behavioral well-being (Rubin, O’Reilly, Luan, & Localio, 2007), was common among both newborn and older infant entrants. In particular, we identified a group of infants (latent group five, 5.6 %) whose care experiences were typified by at least one exit and re-entry to care, with most adopted, with extended family or remaining in care over early childhood, indicating failed re-unification.

Finally, 10 % of newborns spent their first placement in care in a healthcare setting. While some infants may remain on maternity wards until a suitable placement can be found, this finding could suggest a high burden of congenital anomalies, chronic conditions, and preterm birth-related healthcare needs among this group, requiring further investigation.

### 4.2 Comparisons with other countries

Rates of infant entry into care are lower in Australia and Aotearoa New Zealand, and higher in the US, than in England. Previous research suggested that lower rates of infant entry into care in Australia and Aotearoa New Zealand may partly be explained by a greater provision of evidence-based parenting programs and other early intervention services for parents at risk of maltreating their children than here in England (Gilbert et al., 2012), though recent evidence on pre-natal reporting in one Australian state highlights that early intervention does not necessarily translate into better support and improved outcomes for children and their families (Taplin, 2017). Higher rates and steeper increases over time in the US are likely linked to the ongoing opioid epidemic (Williams & Sepulveda, 2019). Comparing our findings to recent evidence on infant entry into care in Scandinavian countries, Rates of infant entry into care were generally lower in Denmark, Finland, Norway and Sweden between 2007 and 2016 than in England (Hestbæk et al., 2020). Crucially, we do not know how much of these inter-country differences are driven by parental characteristics.

Only NSW, Australia had comparable data on early childhood outcomes among infants (newborns only) who entered care (Marsh et al., 2017). In England, adoption is a key pathway for children entering care soon after birth and later in infancy; forced adoption is also far more prevalent in England than any other European country (Fenton-Glynn, 2016). In comparison, only 5.1 % of newborns entering care in NSW between Jan 2006 and Dec 2014 were adopted over this period. However, due to Australia’s history of forced adoptions and the brutal legacy it left among its Aboriginal and Torres Strait Islander communities (Sanders, 2012), most children in Australian state care remain in care through permanent care orders (i.e. long-term foster care) (Australian Institute of Health & Welfare, 2019b). Restoration to parental care was also less common in NSW (6.6 % for newborns) than in England (20 % for newborns, 33 % for older infants) (Marsh et al., 2017). It is unclear, however, whether England’s higher restoration rates indicate greater success in strengthening parenting capacity, or whether there is poorer support for parents during pregnancy and infancy to mitigate entry into care in the first place than in NSW (Zhou & Chilvers, 2010).

### 4.3 Possible mechanisms of increases to infant entries into care in England

One potential explanation for the increased infant entry into care in England are the reported cuts to local authority spending on early family support interventions, youth services, and some public health programs from April 2010 onwards (Kelly, Lee, Sibieta, & Waters, 2018; Mason & Broadhurst, 2020; Webb & Bywaters, 2018). There were also several pivotal reports and changes to legislation over the study period, which influenced practice and likely improved early identification of safeguarding risks among unborn and infant children (Gilbert et al., 2012). These included Lord Laming’s review in 2009, which made several recommendations to improve multi-agency working between children’s services, health services, and the police. In particular, the Laming report emphasized that better identification and sharing of information on parents experiencing mental health problems, substance misuse and domestic violence between children’s services and the police and health services were required (Laming, 2009). Later, in 2011, the Munro review highlighted the negative impact of delaying necessary child protection action for babies and toddlers on early childhood attachment and development (Munro, 2011).

In addition to nationwide changes, there exists considerable regional variation in rates of infant and newborn entry into care (Bilson & Bywaters, 2020; Pearson et al., 2020). This may be partly due to differences in the prevalence of risk factors for child maltreatment between local populations. For example, the North East, which consistently had the highest rates of newborn entry into care over the study period, also had the highest rate of babies born with neonatal abstinence syndrome in 2011 among the English regions, suggesting a higher prevalence of mothers with substance misuse problems (Davies et al., 2016). Regional variation is also partly driven by differences between local children’s services practice and service availability to support parents to improve capacity to parent (All parliamentary group for children, 2018; Mason & Broadhurst, 2020; Pearson et al., 2020).

### 4.4 Strengths and limitations

Our findings build upon existing evidence on infant entries into care in England and uncover trends in their care trajectories over early childhood. A key limitation, however, is that we lacked information on parents and siblings. For example, parental mental health problems, alcohol and drug misuse, and having an older sibling previously placed into care are all key features of infant entry into care in England (W. Marsh & Leamon, 2019). While almost all infants in our subgroup analyses exited care over follow-up, we had limited information to assess the permanency of these exits over time. For example, we lacked data on adoption breakdowns and returns to care later in life for children who exited to live with their birth family. We also lacked data for 2014/15 to 2018/19, though we are currently applying for more data to update our analyses. Finally, we lack information about infants who are known to children’s services but who are cared for via informal arrangements with extended family, which account for most children living with kinship carers in England (Nandy, Selwyn, Farmer, & Vaisey, 2013).

### 4.5 Implications for policy and research

Existing evidence makes a clear case for English pre-birth child protection procedure to be strengthened (Lushey et al., 2018; Marsh, Robinson, Shawe, & Gallagher, 2019; Mason et al., 2019), to improve early intervention, potentially reduce the need for entry into care in infancy, and to reduce risk of local authorities infringing parents’ rights under Article 8 of the EHRC (the right to respect for private and family life) (*R (G) v Nottingham City Council*, 2008). Furthermore, with s 20 arrangements commonly used to place infants, particularly newborns, into care, eligibility for free non-means tested legal aid should be extended to any parent subject to pre-birth child protection procedures to improve legal representation and advocacy for vulnerable families. Many parents of infants who enter care in England are already known to children’s social services, having had previous children placed into care (Bedston et al., 2019; Broadhurst et al., 2017, 2018). Therefore, all local authorities should receive ring-fenced funding to provide support for parents following placement of a child into care, such as the Pause or MPower programs (Cox et al., 2017; McCracken et al., 2017).

Nevertheless, the time constraints of pregnancy and a child’s need for stable and nurturing care to support development over early infancy are often at odds with the timeframes needed for parents to make lasting change to capacity to parent. We urgently need recognition by central and local English government that growing numbers of newborns entering care constitute a public health crisis (Gilbert et al., 2009; O’Donnell, Scott, & Stanley, 2008), requiring an equivalent response via the NHS and local authority public health service commissioning activities. In particular, with parental mental health problems, substance misuse and domestic violence frequently cited as key drivers of child protection intervention (Ward et al., 2012), local authorities need sufficient funding to provide alcohol and drug treatment prevention and treatment services, public mental health, and domestic violence support services, which are not mandatory public health services in England meaning they are vulnerable to defunding and decommissioning (Department for Health & Social Care, 2018, 2019).

To inform an evidence-based public health approach to reducing infant and other entries into care in England, researchers need data on the health and welfare needs of parents whose children enter care (Family Rights Group, 2018; Jay, Woodman, Broadhurst, & Gilbert, 2017). Currently, however, CLA data cannot be linked to external sources, with the exception of children who have entered the state school system (i.e. mostly at age 4 or 5 years) who can be linked to national education data (Jay, Mc Grath-Lone, & Gilbert, 2019; Mc Grath-Lone et al., 2016). The collection of personal identifiers would enable linkage of younger children’s records not only to education data but also health, including parental health (Harron, Gilbert, Cromwell, & Van Der Meulen, 2016), and other data sources. Models exist whereby this can be done safely and securely and whereby researchers only access an anonymized version of the dataset. Doing so would enable researchers to link external information on children and their parents to better understand the wider determinants of care entry as well as longer-term outcomes.

## Role of the funding source

This work was supported by the 10.13039/501100000279Nuffield Foundation [grant number KID/42838]; the 10.13039/501100007155Medical Research Council through the UCL Birkbeck Doctoral Training Partnership [grant number MR/R502248/1]; the National Institute for Health Research (NIHR) Children and Families Policy Research Unit; the NIHR Great Ormond Street Hospital Biomedical Research Centre; and Health Data Research UK. The views expressed are those of the author(s) and not necessarily those of the Nuffield Foundation, the MRC, the NIHR or Health Data Research UK.

## Data access

No additional CLA data are available to share. Data may be obtained from a third party (the Department for Education) and are available for approved research projects on request.

## Declaration of Competing Interest

The authors report no declarations of interest.
