# Supplementary material for: Characterizing newborn and older infant entries into care in England between 2006 and 2014
Source: Child Abuse Negl. 2020 Nov;109:104760. doi: 10.1016/j.chiabu.2020.104760 (PMC7718112; doi:10.1016/j.chiabu.2020.104760)
Supplement: Supplementary file 1 [file mmc1.docx]

**Supplementary appendix**

[Figure S1: A flow diagram of cohort selection from the Children Looked After (CLA) data set 2](#_Toc45186142)

[Table S1: Data sources used for country comparisons of annual rates of newborn and infant entry into care 3](#_Toc45186143)

[Latent class analysis model checks 5](#_Toc45186144)

[Table S2: Annual (Apr-Mar) rates of infant entry into care in England per 10,000 live births, by age group at first entry, from 2006/07 to 2013/14 5](#_Toc45186145)

[Table S3: Annual (Apr-Mar) rates of newborn entry into care in England per 10,000 live births, by region, from 2006/07 to 2013/14 6](#_Toc45186146)

[Table S4: Variation by child’s ethnicity in rate of newborn entry into care in England between April 2008 and March 2014 7](#_Toc45186147)

[Table S5: Categorical measures used in this study 8](#_Toc45186148)

# **Figure S1:** A flow diagram of cohort selection from the Children Looked After (CLA) data set. (TOP) full study cohort. (BOTTOM) subset cohort for exploring care over early childhood.

#
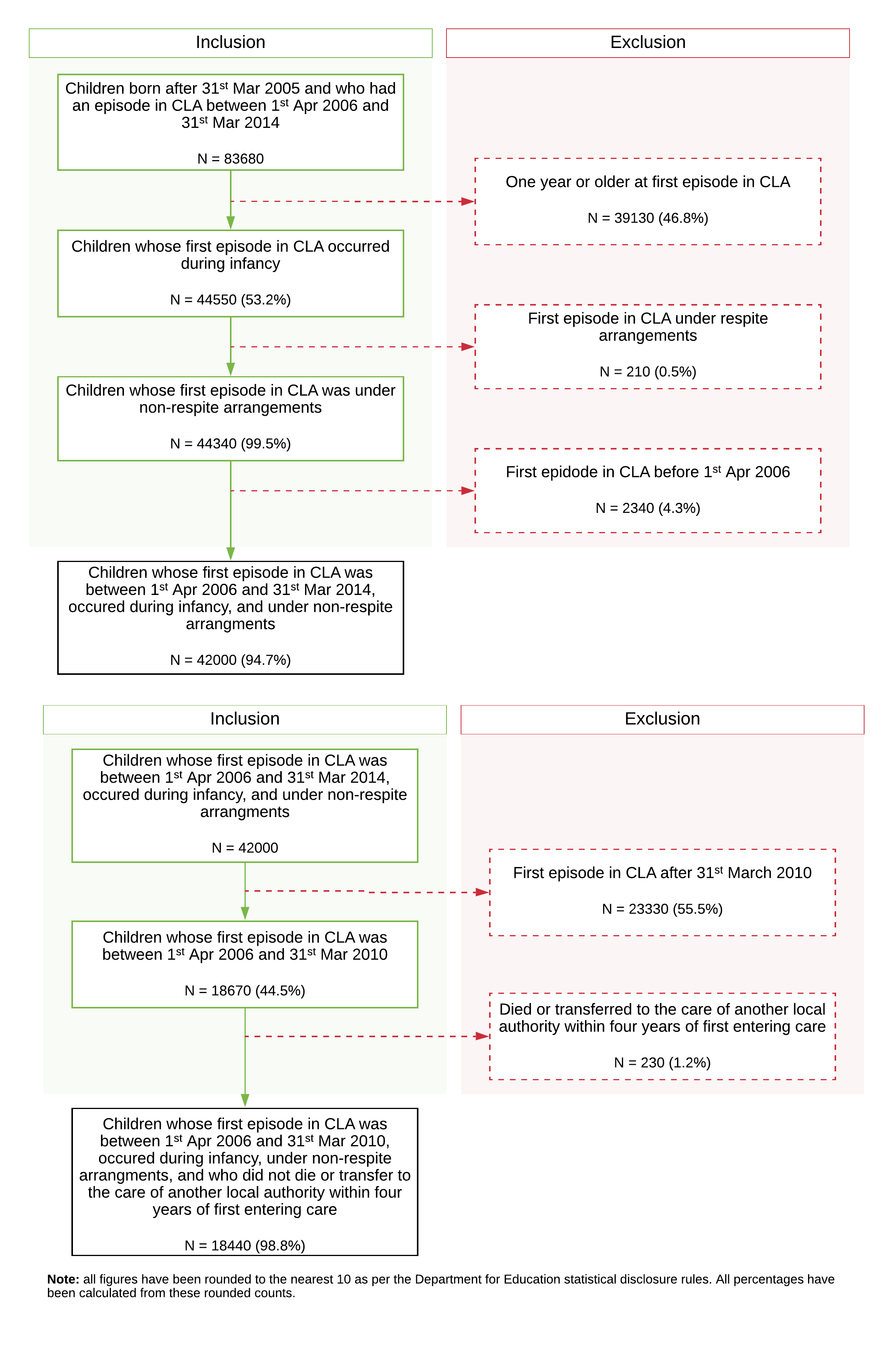


# **Table S1: Data sources used for country comparisons of annual rates of newborn and infant entry into care**

| Country | Newborn | | Infant | | Year |
| --- | --- | --- | --- | --- | --- |
|  | Numerator data source | Denominator data source | Numerator data source | Denominator data source |  |
| Australia | O’Donnell et al, 2019 ‘Infant removals: The need to address the over-representation of Aboriginal infants and community concerns of another ‘stolen generation’’  <https://doi.org/10.1016/j.chiabu.2019.01.017>  Counts from Fig. 3. Removal of infants in the first 7and 31 days (number), Australia. | Australian Bureau of Statistics (dataset) via [http://stat.data.abs.gov.au/Index.aspx?DataSetCode=BIRTHS_MONTH_OCCURRENCE#](http://stat.data.abs.gov.au/Index.aspx?DataSetCode=BIRTHS_MONTH_OCCURRENCE) | <https://www.aihw.gov.au/reports/child-protection/child-protection-australia-2017-18/data>  Table S52: Children admitted to out-of-home care, by age group, states and territories, 2013–14 to 2017–18 (‘<1’ row and ‘Total’ column)  Rates are provided in the table per 1000 children in the population under one year old. | N/A - see numerator description. | July to June |
| England | CLA data extract (current study) supplemented with the reported 2017/18 (April-March) rate of newborn entry into care from <https://doi.org/10.1016/j.childyouth.2020.105164> | <https://www.ons.gov.uk/peoplepopulationandcommunity/birthsdeathsandmarriages/livebirths/datasets/birthsbyareaofusualresidenceofmotheruk> | CLA data extract (current study) supplemented by data on infant entries into care from 2014-2017 https://www.gov.uk/government/collections/statistics-looked-after-children | <https://www.ons.gov.uk/peoplepopulationandcommunity/populationandmigration/populationestimates/datasets/populationestimatesforukenglandandwalesscotlandandnorthernireland> | April to March |
| New South Wales, Australia | <https://doi.org/10.1016/j.childyouth.2017.08.005>  Table 2 (Entered into care counts), reported by calendar year (January-December). | Australian Bureau of Statistics (dataset) via [http://stat.data.abs.gov.au/Index.aspx?DataSetCode=BIRTHS_MONTH_OCCURRENCE#](http://stat.data.abs.gov.au/Index.aspx?DataSetCode=BIRTHS_MONTH_OCCURRENCE) | <https://www.aihw.gov.au/reports/child-protection/child-protection-australia-2017-18/data>  Table S52: Children admitted to out-of-home care, by age group, states and territories, 2013/14 to 2017/18 ((‘<1’ row and ‘NSW’ column)  Rates are provided in the table per 1000 children in the population under one year old. | N/A - see numerator description. | January to December (newborn)  July to June (infants) |
| New Zealand | <https://www.orangatamariki.govt.nz/assets/Uploads/Statistics/data-about-how-we-work-with-children/Babies-and-children-entering-Oranga-Tamariki-care.pdf>  Page 12, top table (unborn and 0-7 categories combined) | Stats NZ Infoshare  <http://archive.stats.govt.nz/infoshare/>  Population > Births > Live births (by sex), stillbirths (Maori and total population) (Annual-Jun) | <https://www.orangatamariki.govt.nz/assets/Uploads/Statistics/data-about-how-we-work-with-children/Babies-and-children-entering-Oranga-Tamariki-care.pdf>  Page 12, top table (all age categories combined) | Stats NZ Infoshare  <http://archive.stats.govt.nz/infoshare/>  Population > Population Estimates > Estimated Resident Population by Age and Sex (1991+) (Annual-Jun)  (Note: we used the ‘Mean Year Ended’ population estimate) | July to June |
| United States | N/A | N/A | Taken from yearly Adoption & Foster Care Statistics (AFCARS) reports between 2006 and 2017 <https://www.acf.hhs.gov/cb/research-data-technology/statistics-research/afcars> | National Center for Health Statistics. Vintage 2018 postcensal estimates of the resident population of the United States (April 1, 2010, July 1, 2010-July 1, 2018), by year, county, single-year of age (0, 1, 2, .., 85 years and over), bridged race, Hispanic origin, and sex. Prepared under a collaborative arrangement with the U.S. Census Bureau. Accessed via CDC wonder (<https://wonder.cdc.gov/>).  For all states, 2006-2017 for <1 year age group | October to September |

# **Latent class analysis model checks**

For our six-class latent class model, all sets of posterior probabilities (i.e. a child’s probability of group membership for each of the six latent groups) for each child contained only one value that was greater than 0.5. After assigning children to latent groups using maximum probability assignment, the average probability of group membership among all children in each latent group was greater than 0.7 for each latent group. This indicated that the six latent groupings were well defined from one another, with no children assigned to more than one group. Further, the odds of correct classification indicated that model assignment to latent groups performed better than chance for each model (Collins & Lanza, 2010).

# **Table S2: Annual (Apr-Mar) rates of infant entry into care in England per 10,000 live births, by age group at first entry, from 2006/07 to 2013/14**

| **Area** | **2006/07** | **2007/08** | **2008/09** | **2009/10** | **2010/11** | **2011/12** | **2012/13** | **2013/14** | **% increase** |
| --- | --- | --- | --- | --- | --- | --- | --- | --- | --- |
| **Newborn (<1 week old)** | 24.9 | 24 | 25.3 | 27.7 | 30.3 | 35.7 | 37.9 | 40.2 | 61.7 |
| **1-3 weeks old** | 14.2 | 13 | 12.6 | 15.8 | 13.7 | 14.5 | 15.6 | 15.3 | 8.4 |
| **4-12 weeks old** | 10.4 | 9.6 | 10.1 | 10.7 | 11.2 | 11.9 | 11.7 | 11.3 | 8.7 |
| **13-25 weeks old** | 8.7 | 8.2 | 9.2 | 10.1 | 9 | 10 | 10.1 | 9.8 | 13.1 |
| **26-38 weeks old** | 5.7 | 6.4 | 6.8 | 7.5 | 6.7 | 6.8 | 6.9 | 7.1 | 24.9 |
| **39-51 weeks old** | 5.3 | 4.9 | 6.1 | 6.4 | 6.4 | 6.1 | 6 | 7.2 | 35.1 |

# **Table S3: Annual (Apr-Mar) rates of newborn entry into care in England per 10,000 live births, by region, from 2006/07 to 2013/14**

| **Area** | **2006/07** | **2007/08** | **2008/09** | **2009/10** | **2010/11** | **2011/12** | **2012/13** | **2013/14** | **% increase** |
| --- | --- | --- | --- | --- | --- | --- | --- | --- | --- |
| **England** | 24.9 | 24.0 | 25.3 | 27.7 | 30.3 | 35.8 | 37.9 | 40.2 | 61.7 |
| **North East** | 41.1 | 40.6 | 36.4 | 60.5 | 51.9 | 65.5 | 72.6 | 79.4 | 93.1 |
| **North West** | 28.5 | 31.4 | 30.6 | 32.0 | 40.4 | 42.8 | 46.0 | 54.4 | 90.8 |
| **Yorkshire and the Humber** | 35.0 | 29.6 | 34.7 | 37.7 | 41.8 | 52.7 | 47.5 | 51.1 | 46.3 |
| **East Midlands** | 21.7 | 26.7 | 22.1 | 24.2 | 32.6 | 36.1 | 39.5 | 43.5 | 100.5 |
| **West Midlands** | 29.6 | 25.7 | 29.3 | 33.8 | 34.7 | 38.3 | 43.3 | 46.4 | 56.9 |
| **East** | 22.4 | 18.8 | 22.3 | 23.8 | 26.0 | 30.1 | 33.5 | 32.3 | 43.8 |
| **London** | 17.4 | 18.3 | 20.4 | 18.6 | 16.5 | 23.3 | 22.4 | 22.6 | 30.1 |
| **South East** | 18.3 | 19.8 | 21.2 | 23.2 | 26.3 | 28.9 | 31.5 | 32.3 | 76.8 |
| **South West** | 21.9 | 21.1 | 22.1 | 24.0 | 26.6 | 36.2 | 39.3 | 39.2 | 78.7 |

# **Table S4: Variation by child’s ethnicity in rate of newborn entry into care in England between April 2008 and March 2014**

| Year (Apr-Mar) | Ethnic Group | Raw counts | | | | Rate of newborn entry into care per 10,000 live births | | |
| --- | --- | --- | --- | --- | --- | --- | --- | --- |
|  |  | Non-missing ethnicity | | Missing ethnicity | | Using non-missing data only | Taking into account missing data | |
|  |  | Number of newborns entering care | Live births* | Number of newborns entering care | Live births* | Observed | Lowest possible | Highest possible |
| 2008/09 – 2009/10 | Asian | 120 | 131311 | 50 | 94973 | 9.14 | 5.3 | 12.95 |
|  | Black | 180 | 72194 |  |  | 24.93 | 10.77 | 31.86 |
|  | Other | 460 | 93044 |  |  | 49.44 | 24.47 | 54.81 |
|  | White | 2760 | 951359 |  |  | 29.01 | 26.38 | 29.54 |
|  |  |  |  |  |  |  |  |  |
| 2010/11 – 2011/12 | Asian | 110 | 141238 | 110 | 45709 | 7.79 | 5.88 | 15.58 |
|  | Black | 170 | 72602 |  |  | 23.42 | 14.37 | 38.57 |
|  | Other | 600 | 101124 |  |  | 59.33 | 40.86 | 70.21 |
|  | White | 3550 | 1011801 |  |  | 35.09 | 33.57 | 36.17 |
|  |  |  |  |  |  |  |  |  |
| 2012/13 – 2013/14 | Asian | 140 | 146058 | 260 | 41254 | 9.59 | 7.47 | 27.39 |
|  | Black | 160 | 69850 |  |  | 22.91 | 14.4 | 60.13 |
|  | Other | 700 | 106857 |  |  | 65.51 | 47.26 | 89.84 |
|  | White | 4030 | 983676 |  |  | 40.97 | 39.32 | 43.61 |

Lowest possible = number with non-missing ethnicity*10000/(live births with non-missing ethnicity+ live births with missing ethnicity)

Highest possible = (number with non-missing ethnicity + number with missing ethnicity)*10000/live births with non-missing ethnicity

* Data on live births stratified by child’s ethnicity taken from ONS user requested data (request number: 006134): <https://www.ons.gov.uk/peoplepopulationandcommunity/birthsdeathsandmarriages/livebirths/adhocs/006134birthsbyethnicitysexregionandimdquintilebyfinancialyear2007to2013> (last accessed 18/09/2020)

# **Table S5: Categorical measures used in this study**

| Measure name | Description | Notes |
| --- | --- | --- |
| Legal route of entry into care | Care Order | A Care order transfers parental responsibility to the local authority |
|  | Child Assessment Order | Child assessment orders requires parents to make a child available for assessment by the local authority or another third party, where it is suspected that the child is at risk of significant harm. |
|  | Emergency child protection | Entry under emergency protection orders (child can enter care for up to 8 days) and police protection powers (child can enter care for up to 72 hours). |
|  | Placement Order | These orders ‘free’ a child in local authority care for adoption. |
|  | Supervision Order | A supervision order grants the local authority powers to advise, help and befriend (i.e. monitor) a child to promote their health and wellbeing. |
| Placement type | stranger foster care | Placement with foster carers (not known to family or child) |
|  | kinship foster care | Placements with extended family members or with family friends |
|  | healthcare setting | NHS/Healthcare trust providing medical/nursing care |
|  | with parents at home | i.e. under a supervision or child assessment order |
|  | family or mother/baby placement | Placement outside of the home with one or both parents. This includes placement in mother-baby units. |
|  | other placement type | Includes placement with approved adopters, in residential children’s homes and other (unspecified). |
| Primary category of need | N1 – Abuse and Neglect | The primary categories of need are hierarchical where ‘Abuse or Neglect’ is the highest in the hierarchy (i.e. if multiple categories of need are identified then only the highest in the hierarchy will be recorded) |
|  | N2 – Child’s disability |  |
|  | N3 – Parental illness or disability |  |
|  | N4 – Family in acute stress |  |
|  | N5 – Family dysfunction |  |
|  | N6 – Socially unacceptable behaviour |  |
|  | N7 – Low income |  |
|  | N8 – Absent parenting |  |
| Exits from care | Any exit | Any exit from care indicated in their CLA record. |
|  | Exit but later re-entered care | Indicated where a child exited care and subsequently had a further placement in care. |
|  | Restored to parental care | The child exited care to return home to live with their parent(s). |
|  | Exit to live with extended family | The child exited care under a Special Guardianship Order or Residence Order (replaced by the Child Arrangements Order in April 2014) |
|  | Exit to adoption | The child exited care due to an adoption order. |
